# Cognitive changes following multiple-modality exercise and mind-motor training in older adults with subjective cognitive complaints: The M4 study

**DOI:** 10.1371/journal.pone.0196356

**Published:** 2018-04-26

**Authors:** Narlon Cassio Boa Sorte Silva, Dawn P. Gill, Adrian M. Owen, Teresa Liu-Ambrose, Vladimir Hachinski, Ryosuke Shigematsu, Robert J. Petrella

**Affiliations:** 1 School of Kinesiology, Faculty of Health Sciences, Western University, London, Ontario, Canada; 2 Lawson Health Research Institute, London, Ontario, Canada; 3 Graduate Program in Health & Rehabilitation Sciences, Faculty of Health Sciences, Western University, London, Ontario, Canada; 4 School of Health Studies, Faculty of Health Sciences, Western University, London, Ontario, Canada; 5 Centre for Studies in Family Medicine, Department of Family Medicine, Western University, London, Ontario, Canada; 6 Canadian Centre for Activity and Aging, Western University, London, Ontario, Canada; 7 The Brain and Mind Institute, Department of Psychology, Western University, London, Ontario, Canada; 8 Department of Physical Therapy, Faculty of Medicine, University of British Columbia, Vancouver, British Columbia, Canada; 9 Djavad Mowafaghian Centre for Brain Health, Vancouver, British Columbia, Canada; 10 Department of Clinical Neurological Sciences, London Health Sciences Centre, Western University, London, Ontario, Canada; 11 Faculty of Education, Mie University, Tsu, Japan; Nathan S Kline Institute, UNITED STATES

## Abstract

**Background:**

We investigated the effects of multiple-modality exercise with additional mind-motor training on cognition in community-dwelling older adults with subjective cognitive complaints.

**Methods:**

Participants (n = 127, mean age 67.5 [7.3] years, 71% women) were randomized to receive 45 minutes of multiple-modality exercise with additional 15 minutes of either mind-motor training (M4, n = 63) or control (balance, range of motion and breathing exercises [M2, n = 64]). In total, both groups exercised 60 minutes/day, 3 days/week, for 24 weeks. Standardized global cognitive functioning (GCF), concentration, reasoning, planning, and memory were assessed at 24 weeks and after a 28-week no-contact follow-up.

**Results:**

There were no significant differences in the study primary outcomes. The M4 group, however, showed trends for greater improvements in GCF and memory (both, *P* = .07) compared to the M2 group at 24 weeks. Significant differences between group in GCF (*P* = .03) and memory (*P* = .02) were observed after the 28-week no-contact follow-up favouring the M4 group.

**Discussion:**

Additional mind-motor training did not impart immediate greater benefits to cognition among the study participants.

## Introduction

Findings from laboratory work and clinical trials for the treatment of dementias (e.g., Alzheimer’s disease) have consistently produced disappointing results, with the possibility of a single cure being very unlikely [1,2]. As a result, strategies to prevent and treat cognitive decline early in life have gained increased attention [2]. Indeed, the focus of research has started to shift from stages in which the disease has been established to preclinical or even asymptomatic stages [3]. This shift is extremely important since underlying pathophysiological process of dementia may take place decades before disease diagnosis occur [3]. In this perspective, the identification of biomarkers and the management of modifiable risk factors seem to be of greatest priority [2,4]. Of particular interest, cognitively healthy older adults with subjective cognitive complaints (SCC) [5] may represent a portion of the population experiencing early signs of cognitive decline due to underlying pathological changes before the onset of clinical impairment [6,7]. Although these individuals demonstrate preserved cognitive function in traditional neuropsychological tests—therefore, not meeting the criteria for mild-cognitive impairment (MCI) or dementia—they often report cognitive complaints relating to worsening of memory and thinking skills [8]. In fact, SCC has been associated with poorer scores in objective cognitive assessments [9], the establishment of clinical impairment nearly 2 decades after first report [10], and greater health care utilization [11]. More strikingly, older adults with SCC show patterns of cortical and hippocampal atrophy similar to that of patients with the diagnosis of MCI [12]. These observations suggest that older adults with SCC compose an ideal target group for early-in-life intervention programs aiming at mitigate cognitive impairment, which could culminate in the best clinical outcomes [2,13] and alleviate burdens on the health care systems worldwide [11].

Habitual participation in aerobic exercise (AE) interventions alone [14] or combined with mind-motor training [15] appears to benefit cognition in individuals without known cognitive impairment and in those with dementia [16]. Despite promising evidence, the impact of AE on cognitive function in the aging population remains equivocal [17], particularly in those with SCC. Colcombe and Kramer [18] conducted a meta-analysis of 18 interventions and found a significant effect of AE on cognition, with a greater effect on executive functioning. Colcombe et al [19] also observed improvements in brain plasticity after 6 months of progressive AE compared to a stretching group. Similarly, after 6 months of a moderate-intensity exercise program, Lautenschlager et al [20] observed improved cognitive scores in older adults with cognitive impairment compared to a usual care group. Erickson et al [21] found that following a 12-month moderate-intensity AE regimen, healthy older adults showed growth in volume in anterior hippocampal regions, while hippocampal atrophy was seen over the same period in the active control group. Smith et al [22] observed improvements in neural efficiency during semantic memory retrieval tasks in older adults with MCI following a 12 week moderate intensity, treadmill-based AE regimen. Finally, Ten Brinke et al [23] found that 6-months of moderate intensity, walking-based exercise increased hippocampal volume among older adults with probable MCI compared to a balance and toning control.

Although AE training is related to improvements in cognition, a recent Cochrane review suggests there is insufficient evidence to conclude that cognitive improvements are solely attributable to improved cardiovascular fitness [24]. As well, findings from other meta-analytic studies indicate lack of consistency across different exercise studies, which could mostly be due to variability in cognitive tests applied, sensitivity of cognitive tests to detect treatment effects, and cognitive and physical health at baseline [17,25]. Furthermore, several aspects of these investigations may raise concerns regarding the feasibility of exercise protocols administered in such laboratory settings (i.e., real-world applicability and translation to community settings). Moreover, most studies have failed to comply with current guidelines for exercise in older adults with regards to exercise type, intensity, frequency and duration [26]. These guidelines also emphasize the importance of multiple-modality exercise programs over single-modality exercise programs to enhance overall health and quality of life in the general population of older adults [26,27], although evidence is still limited in more specific groups (e.g., individuals with SCC). The evidence is even more scarce with regards to multiple-modality exercise interventions and cognitive function in older adults at risk for dementia. As such, more research is warranted. In addition, from a clinical and scientific perspective, exploring the combination of multiple-modality exercise with alternative, and perhaps more feasible (e.g., group-based, low-cost, and easily administered), forms of mind-motor training (simultaneous cognitive and physical engagement) on cognitive outcomes may provide further support for optimal exercise interventions to improve overall health and promote additive benefits to cognitive function in older adults at risk for cognitive impairment [26].

Square-stepping exercise (SSE) [28] is a novel form of mind-motor training that has been associated with positive effects on global [29] and domain-specific cognitive functioning [29,30] in older adults. Although no investigation on the specific physiological mechanisms were conducted in these studies, we postulate that these improvements could be attributable to increased neuroflexibility and/or plasticity, which in turn is a result of exercise-induced synaptogenesis and angiogenesis in the brain, particularly in brain regions associated with executive function and working memory[31,32]. The SSE program is a simple, low-cost, indoor, group-based exercise program designed to improve fitness of the lower extremities and serve as a strategy to prevent falls in older adults [28,33]. Results from short-term studies [28,33] showed that the SSE was equally as effective as strength training and more effective than a weekly walking session to improve lower-extremity function and reduce fall risk factors. Although the impact of SSE on cognitive function remains relatively unknown, pilot work suggests the potential for SSE to benefit cognition. Teixeira et al [29] observed improvements in global cognition, attention, and mental flexibility among cognitively healthy older adults following 16-weeks of SSE. These findings were advanced by Shigematsu et al [30] who investigated the cumulative impact of SSE training over 6 months on cognition among non-demented, community-dwelling older adults. Although improvements in memory were observed following both training regimens, improved executive functioning was reserved for those performing SSE on a weekly basis. Moreover, SSE is an innovative, inexpensive, and easily employed group exercise program, lending itself as a mind-motor task that may be easily incorporated into standard exercise programs for older adults.

There is some research to support the notion that aerobic and other forms of exercise may impart improvements to cognition in older adults; as well, the preliminary findings suggest the potential utility of SSE as an exercised-based cognitive intervention; it remains unclear, however, whether older adults showing signs of early cognitive deterioration are susceptible to improvements in cognition following multiple-modality exercise with additional mind-motor training. Thus, we investigated the effects of group-based based, multiple-modality exercise with additional SSE would in cognition compared to multiple-modality exercise alone in older adults with SCC living in the community.

## Methods

### Study design

The M4 Study was a two-arm randomized controlled trial (RCT) implementing a 24-week intervention program with a 28-week no-contact follow-up [34]. Assessments were performed at baseline, 24 weeks (intervention endpoint) and 52 weeks (study endpoint). After baseline assessments, participants were randomized to either the multiple-modality exercise with mind-motor training intervention group (Multiple-Modality, Mind-Motor [M4]) or to the multiple-modality exercise active control group (Multiple-Modality [M2]). The randomization sequence was computer generated, and concealed envelopes were used to assign group status. All assessors were blinded to group assignment.

### Participants

Details of the M4 study participants and eligibility criteria have been published [34,35]. Briefly, the study included community-dwelling older adults aged 55 years or older, who self-reported a cognitive complaint (defined answering positively to the question “Do you feel like your memory or thinking skills have got worse recently?”) [36]. Subjective cognitive complaints are defined as a subjective perception of cognitive deterioration by an individual or their peers, even though the individual may seem to perform well in neuropsychological tests, and may not demonstrate signs of objective cognitive impairment [13,37,38]. As well, we included individuals who were fully independent in functional activities (maximum score in the Lawton-Brody Instrumental Activities of Daily Living scale [8/8]) [39]. Individuals were excluded if they had diagnosis of dementia and/or scored < 24 on the Mini-Mental State Examination (MMSE) [40], had major depression, recent history of severe cardiovascular conditions, any neurological and/or psychiatric disorders, or were unable to comprehend the study letter of information.

The study was registered with ClinicalTrials.gov on 29 April 2014 (Identifier: NCT02136368). The Western University Health Sciences Research Ethics Board approved this project and all participants provided written informed consent prior to taking part in the study.

### Multiple-modality exercise intervention

Participants in both groups received 45 minutes of group-based, standardized, multiple-modality exercise, described in detail elsewhere [34]. The M4 group performed an additional 15 minutes of mind-motor training (i.e., SSE), whereas the M2 group underwent 15 minutes of active control condition focused on balance, range of motion and breathing exercises. In total, participants in both groups exercised 60 minutes/day, 3 days/week for 24 weeks.

The multiple-modality exercise intervention incorporated a 5-minute warm-up, 20-minute AE, 5-minute cool down, followed by 10 minutes of resistance training (see [41]) and 5 minutes of stretching. We prescribed AE intensity via target heart rates (HR) determined at baseline using the STEP^™^ tool [42]. During the AE component, participants were encouraged to keep their HR at 65–85% of their predicted maximum HR (HRmax) and/or at a rating of 5–8 on the 10-point modified Borg Rating of Perceived Exertion (RPE) scale [27]. We conducted HR monitoring part way through and at the end of the AE component during each exercise session. Participants were instructed to record HR and RPE immediately after each monitoring in a training log provided by the research team. Target HR was recalculated at 12 weeks to adjust for short-term cardiorespiratory adaptations.

#### Active control intervention

The active control group underwent additional 15 minutes of balance, range of motion and breathing exercises, prior to the 5 minutes of stretching. This component of the intervention was focused on low-intensity exercises without use of any additional loading (e.g., hand weights or resistance bands), with HR maintained below target zone, and was deemed as a suitable active control condition, as these exercises have not been found to impart cognitive benefits [34]. Participants performed 10 minutes of static (e.g., postures in narrow stance, tandem stance and single leg stance), dynamic (e.g., walk tandem line on heels or toes) and functional balance (e.g., changing direction on cue, walking with head turns). The session ended with 5 minutes of range of motion exercises (e.g., shoulder, hip and wrist circles) and accompanied by either standing or sitting breathing exercises.

#### Mind-motor training intervention

**Fig 1:**
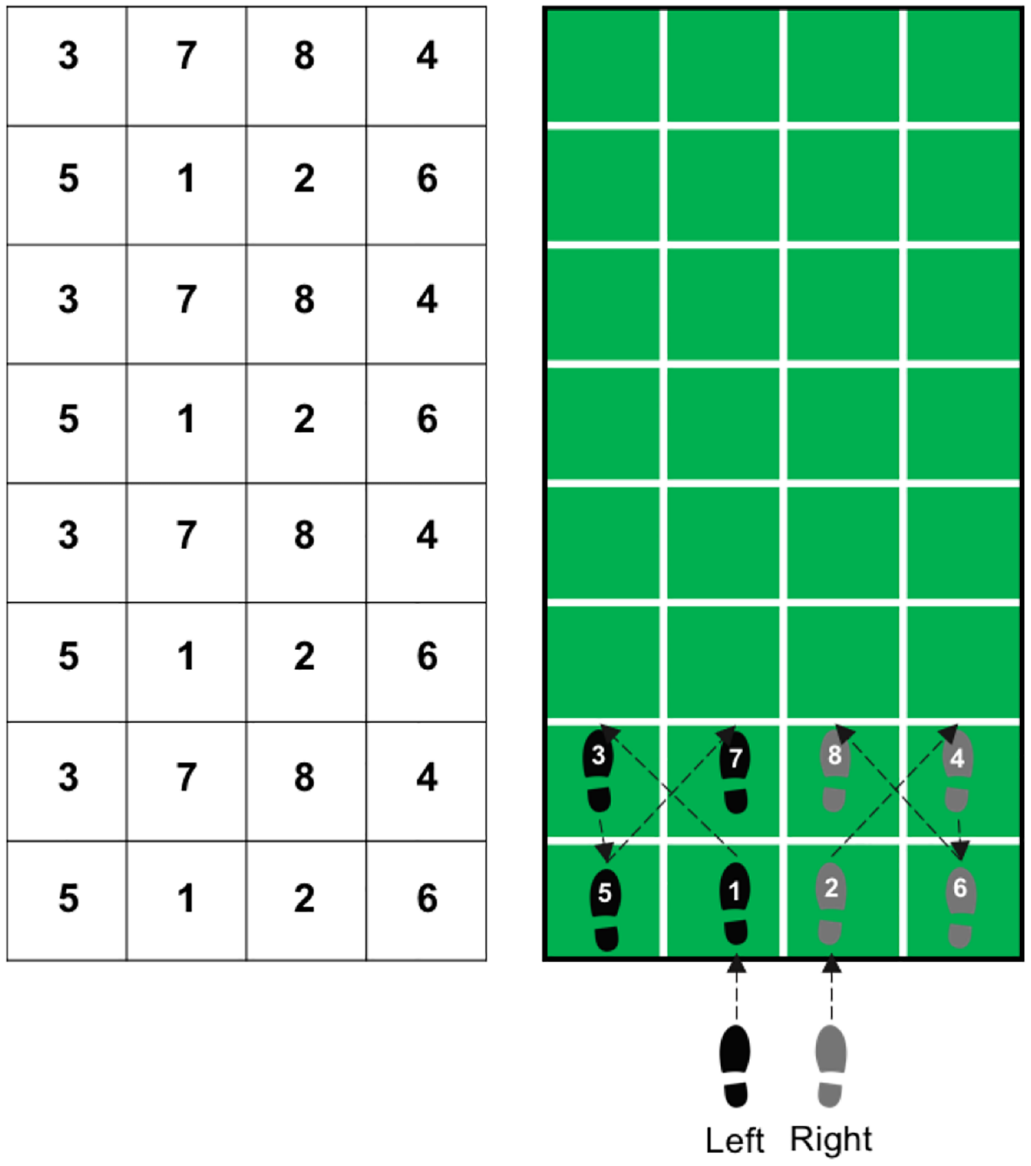
Square-stepping exercise. Illustration of the square-stepping exercise training protocol. The numbers indicate the order in which the steps are performed, the arrows indicate the sequence.

In addition to the multiple-modality exercise intervention, participants within the M4 group also performed SSE training [28], prior to the 5 minutes of stretching. The SSE program is a group-based intervention performed on a gridded floor mat (2.5 m × 1 m) containing 10 rows with 4 equal-sized squares per row. The training protocol entails the reproduction of previously demonstrated complex stepping patterns on the SSE mat (see Fig 1). The stepping patterns are demonstrated by an instructor and participants are expected to memorize, and further attempt to reproduce each stepping pattern by memory. Instructors could not physically intervene, but in instances where participants were having difficulty reproducing the SSE patterns, they were provided oral cues. There are more than 200 stepping patterns created for SSE [28], and the complexity of these stepping patterns is given according to the number of steps per pattern, as well as the order and direction of foot placement across the SSE mat. In our study, the SSE sessions were carried out in groups of no more than 6 participants per mat. To ensure equal group progression throughout the program, the complexity of the stepping patterns within each session was increased only when the majority of participants (i.e., 75%) had successfully performed a given stepping pattern at least four times. The goal was to progress through as many SSE patterns as possible over the 24-week intervention period. Additionally, to create a positive social atmosphere, participants were encouraged to assist each other, as necessary, by providing cues to accurately perform the stepping patterns.

### Study assessments

#### Descriptive variables

Baseline assessments were performed after obtaining written informed consent and prior to participant randomization. Neuropsychological assessments were performed using the MMSE, the Montreal Cognitive Assessment (MoCA), [43] and the Centre for Epidemiological Studies Depression Scale [44]. Participant clinical and demographic data included: age, sex, race, medical history, weight, height, body mass index, and 24-hour blood pressure. Additionally, we assessed cardiorespiratory fitness (predicted maximal oxygen consumption [pVO_2_ max]) at baseline, and again 24 and 52 weeks for further exploratory analyses using the STEP tool [42].

#### Cognitive outcome

Outcome assessment was performed at baseline, 24 weeks (intervention endpoint) and 52 weeks (after a 28-week no-contact follow-up) using the Cambridge Brain Sciences (CBS) computerized cognitive battery [45] (https://www.cambridgebrainsciences.com/). The CBS contains 12 non-verbal cognitive tasks that cover four broad cognitive domains (i.e., concentration [3 tasks], reasoning [3 tasks], planning [2 tasks], and memory [4 tasks]) and correlates highly with measures of general fluid intelligence [46] (see [34] for full CBS description). These tasks are fully automated, and have been used to effectively evaluate cognition in several large-scale, population-based studies [45]. It is an adaptive testing platform that randomly generates novel versions of the tasks between individual trials and can be administered within 60 minutes, thereby, it is believed that the CBS can minimize practice effects and participant fatigue compared to paper-based neuropsychological assessments.

The CBS was administered on the first day of assessments for familiarization purposes (short version) and re-administrated on the second day of assessments for data collection (full version). We used data gathered from participants’ performance in the CBS to create composite scores [47]. These composites scores were derived by first converting all individual outcomes from the CBS tasks to standardized *z* scores. Next, standardized scores were averaged within each one of the four cognitive domains, then domain-specific composite scores were averaged to create a global cognitive functioning (GCF) score, ensuring that the four cognitive domains were weighted equally.

The study primary outcome was differences between groups in estimated mean change from baseline to 24 weeks in GCF. Secondary outcomes included changes at 52 weeks in GCF and changes in composites scores of concentration, reasoning, planning, and memory at 24 and 52 weeks.

### Sample size

Results from a previous meta-analysis indicated that exercise would have an overall effect on cognition with a moderate effect size (*d = 0*.*48*) [18]. No study to date, however, has observed the effect of a 24-week multiple-modality exercise program with mind-motor training on GCF in community-dwelling older adults. In addition, although the CBS is grounded in well-validated neuropsychological tests [45], it has not been used to date as an outcome in published exercise intervention studies. For these reasons, sample size for the proposed study was approximated by using the effect size approach, combined with feasibility and comparisons to sample sizes used in other similar studies [20,36]. Hence, we determined that a sample size of 52 participants per group would have an 80% power at the 5% significance level to detect an effect size of *d =* 0.55. Considering a dropout rate of 20%, our final sample size was estimated at 65 participants per group.

### Statistical analysis

We conducted linear mixed models for repeated measurements [48] to assess differences between groups in mean change from baseline to 24 weeks. Within the models, we also examined differences between groups from baseline to 52 weeks, and differences within groups from baseline to 24 and 52 weeks. The terms included in the models were: group, time, and group × time. Time was modeled categorically using two indicator variables representing each time point (baseline as reference category). All analyses were performed using the intent-to-treat approach, including all randomized participants, regardless of compliance with the program and follow-up assessments [48]. An advantage of the mixed effects regression modeling approach is that it does not require each participant to have the same number of measurements provided data are missing at random (i.e., after taking observed data into account, there are no systematic differences between participants with complete data as compared to those with missing data). This is also an assumption made by most multiple imputation methods [48]. We also performed a sensitivity analysis including only those who completed the study assessments at all time points. As well, for the main outcomes of the study, we conducted analyses adjusting for global cognitive functioning at baseline (MoCA scores). Interpretation of study results were primarily based on mean estimation and associated 95% confidence intervals.

Finally, additional analyses were conducted using linear regression models to investigate whether change in cardiorespiratory fitness (pVO_2_max) would be associated with change in the study outcomes, following previous methods [21,49]. For this purpose, change scores from baseline to 24 and 52 weeks for all cognition outcomes as well as for VO_2_max were calculated and included in the models adjusting for age, gender and years of education. If pVO_2_max significantly predicted changes in cognition, a mediation effect would be assumed. All analyses were performed using IBM^®^ SPSS^®^ Statistics for Mac, Version 21 (Armonk, NY: IBM Corp).

## Results

### Enrollment, randomization, and adherence

**Fig 2:**
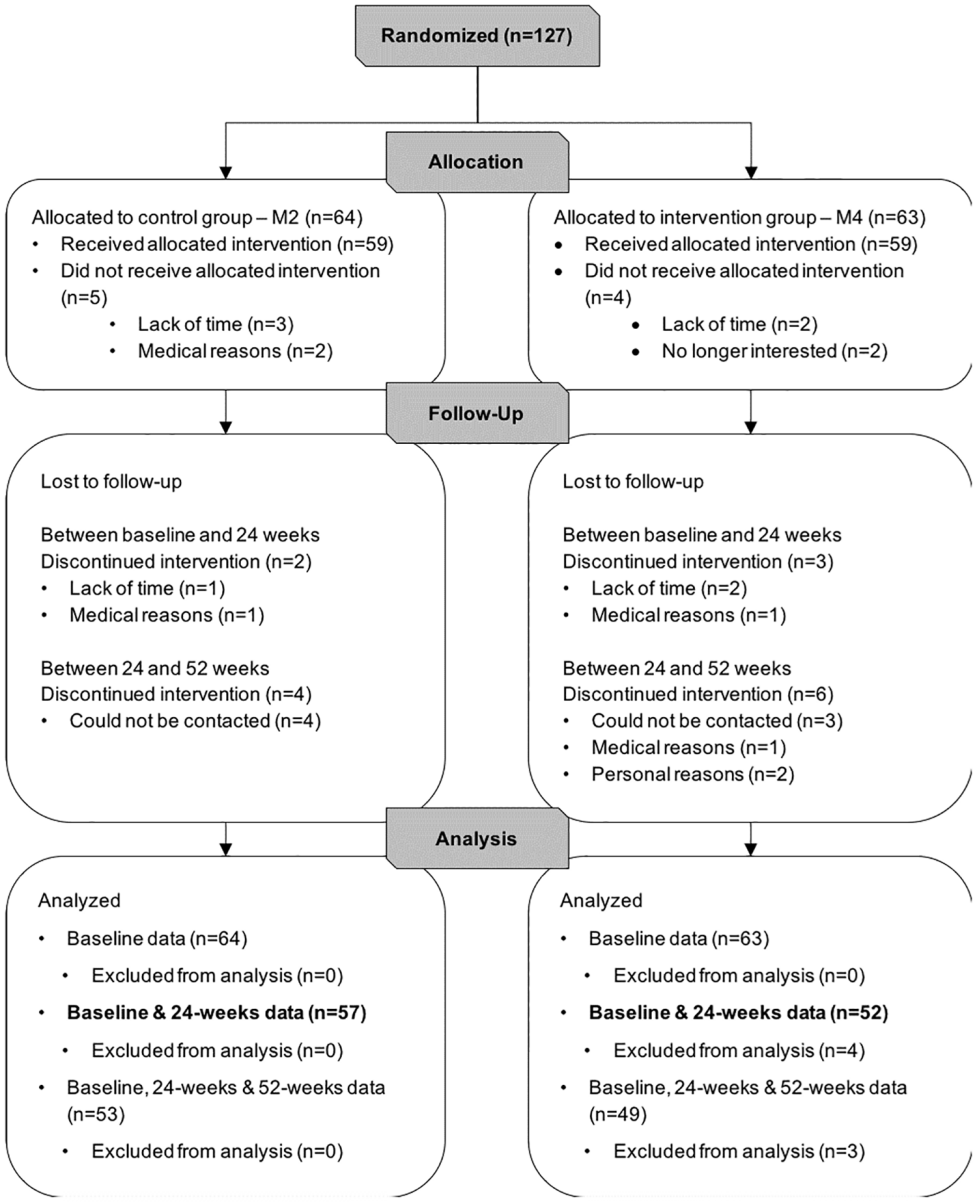
Flow of participants. Flow of participants in the 24-week randomized controlled trial with a 28-week no-contact follow-up. For the M4 group, data from 4 participants were missing at 24 weeks and, therefore, not included in analyses.

This study was conducted between January 13, 2014 and March 14, 2016. Participants were enrolled in 4 waves of assessments and intervention over a period of 14 months. During the screening process, 169 individuals were assessed for eligibility; 11 did not meet the inclusion criteria and 31 declined to participate. Thus, 127 participants were included and randomized to either the M2 (n = 64) or M4 (n = 63) groups, 109 participants attended assessments at 24 weeks, and 102 returned for the final assessments at 52 weeks (see Fig 2). Participants had completed the study and the average attendance to the exercise sessions was 72% for the M2 group (52 out of 72 sessions) and 68% for the M4 group (49 out of out of 72 sessions). A two-sided independent samples t-test revealed no significant differences between groups in participant average attendance (p = .3). At the end of the intervention, participants in the M4 group had achieved the Advanced Level 3 of the SSE program, with stepping patterns ranging from 12 to 16 steps, and with steps performed in a broad range of directions (backwards, diagonal, and backwards diagonal), as well as with stepping patterns incorporating wide and long steps (3 to 5 squares between feet). Considering attendance level and program achievement, the SSE program was shown to be feasible in this specific population (i.e., older adults with SCC) and no study-related adverse events were recorded.

Table 1 provides the baseline descriptive characteristics of the 127 participants. In overall, the study participants were mostly Caucasian, highly educated and presented with signs of cognitive deterioration based on mean MoCA scores. Further observation of the domain-specific MoCA scores revealed that participants in both groups showed low scores in the delayed-recall memory composite, which indicate memory loss possibly underlying the nature of the self-reported SCC. As well, even though participants involved in the study were high-functioning and lived independently in the community, pVO_2_max assessment yielded classification of ‘poor’ to ‘fair’ cardiorespiratory fitness compared to age and gender reference values [50].

**Table 1:** Baseline characteristics of study participants by randomization group.

Variables	M2 (n = 64)	M4 (n = 63)
Demographics		
Age, yr	67.4 (7.2)	67.6 (7.5)
Women	46 (71.9%)	44 (69.8%)
Caucasian	62 (98.4%)	61 (96.8%)
Education, yr	13.8 (3)	13.3 (2.7)
MoCA, score	25.6 (2.4)	25.3 (2.7)
MMSE, score	29.2 (1)	29 (1.2)
CES-D, score	9.4 (7.4)	10 (8.9)
24-hour systolic BP, mmHg	129.6 (15.2)	126.5 (11.3)
24-hour diastolic BP, mmHg	74.2 (8.3)	72.2 (8.1)
Weight, kg	80.8 (17.7)	80 (13.8)
Height, m	1.65 (0.1)	1.65 (0.1)
BMI, kg/m^2^	29.7 (6.2)	29 (4.1)
pVO_2_max, ml/kg/min	26.8 (8)	27.1 (7.9)
Medical history, *n* (%)		
Hypertension	32 (50%)	36 (57.1%)
Hypercholesterolemia	23 (35.9%)	28 (44.4%)
Type 2 diabetes	5 (7.8%)	7 (11.1%)
Myocardial infarction	4 (6.3%)	5 (7.9%)
Atrial fibrillation	-	3 (4.8%)
Angina/coronary artery disease	1 (1.6%)	2 (3.2%)
Aneurysm	1 (1.6%)	2 (3.2%)
Former smoker	28 (44.4%)	29 (46%)
Current smoker	1 (1.6%)	1 (1.6%)
Study outcomes, z scores		
GCF	.058 (.638)	–.047 (.687)
Concentration	.008 (.788)	–.008 (.746)
Reasoning	.041 (.707)	–.041 (.838)
Planning	.091 (.76)	–.092 (.96)
Memory	.091 (.824)	–.047 (.803)

NOTE: Data presented either as mean (standard deviation) or no. (%) where applicable.

Abbreviations: GCF, global cognitive functioning; M2, multiple-modality group; M4, multiple-modality, mind-motor group; MMSE, Mini-Mental Status Examination; MoCA, Montreal Cognitive Assessment; CES-D, Centre for Epidemiological Studies Depression Scale; BP, blood pressure; pVO_2_max, predicted maximal oxygen consumption.

### Study outcomes

**Fig 3:**
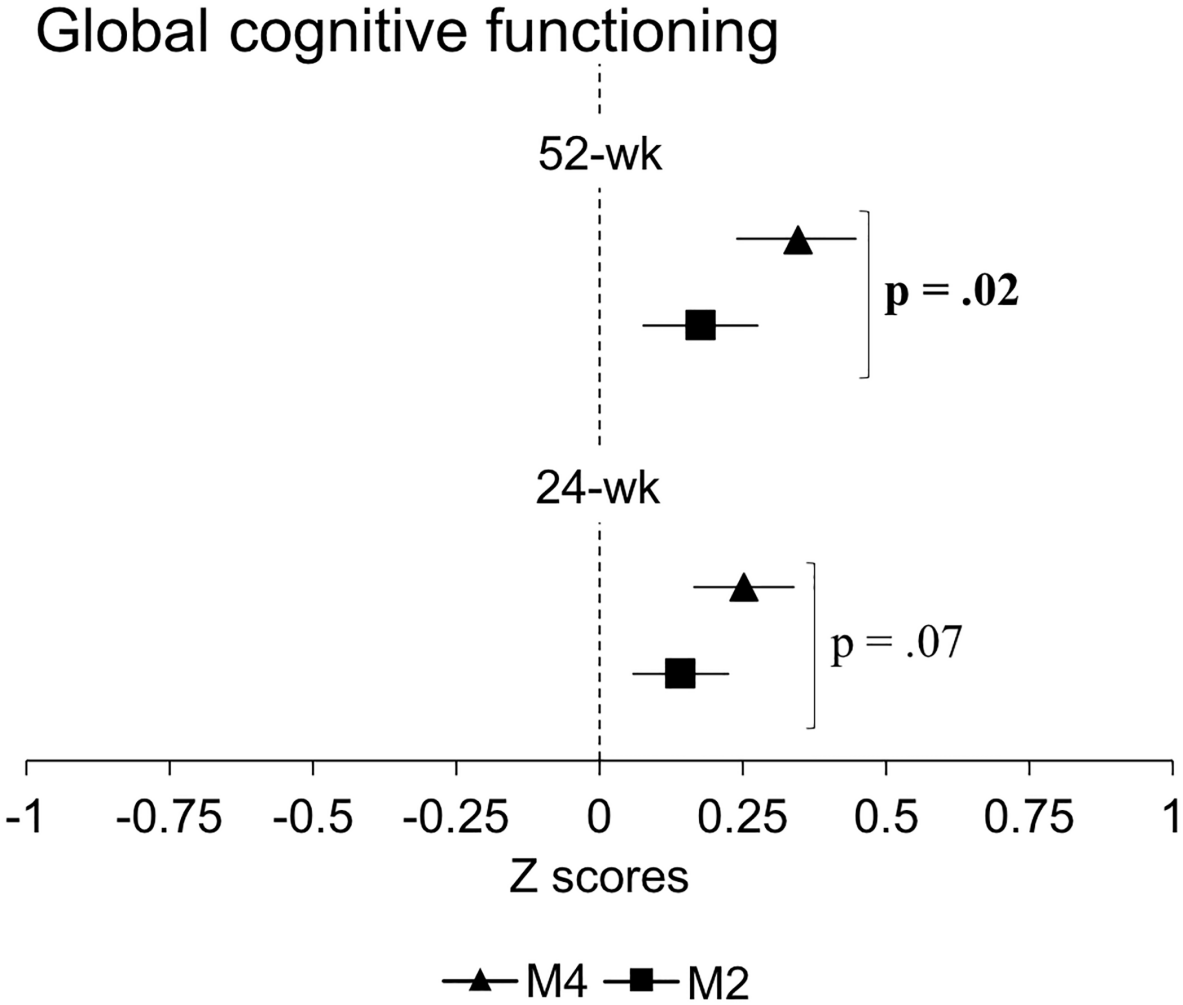
Changes in global cognitive functioning. Solid squares (M2) and triagles (M4) represent point estimated group mean change from baseline; bars represent associated 95% confidance intervals. P value indicates significant differences between groups in estimated mean change from baseline. Abbreviations: M2, multiple-modality group; M4, multiple-modality, mind-motor group. 24-wk, intervention endpoint; 52-wk, study endpoint.

**Fig 4:**
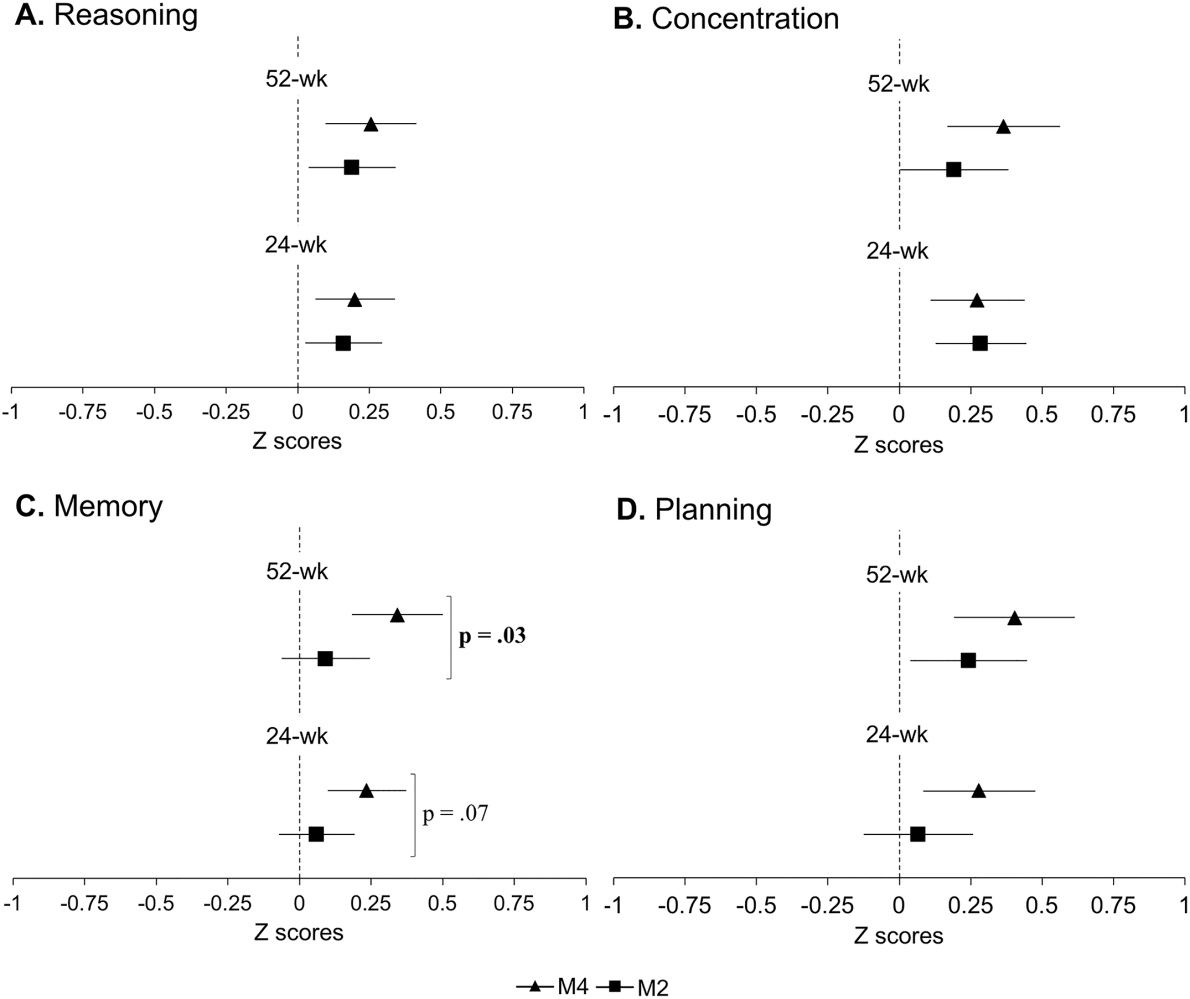
Changes in domain-specific cognitive function. Solid squares (M2) and triagles (M4) represent point estimated group mean change from baseline; bars represent associated 95% confidance intervals. P value indicates significant differences between groups in estimated mean change from baseline. Abbreviations: M2, multiple-modality group; M4, multiple-modality, mind-motor group. 24-wk, intervention endpoint; 52-wk, study endpoint.

At 24 weeks, no significant differences between groups in estimated mean change from baseline were observed for any outcomes (Table 2). The M4 group, however, demonstrated trends for greater improvements in GCF (p = .07) and memory (p = .07) compared to the M2 group. Although there were only trends for statistically significant differences between groups, both groups demonstrated improvements in GCF (Fig 3), concentration and reasoning, and the M4 group also showed improvements in planning and memory at 24 weeks (Fig 4). At 52 weeks, the M4 group showed greater GCF (p = .02) and memory (p = .03) scores compared to the M2 group (Table 2). Both groups also retained improvements in GCF (Fig 3), concentration, reasoning and planning, and the M4 group retained improvements in memory (Fig 4). Complete case analysis resulted in similar findings to those from the intent-to-treat analysis (Table 2).

**Table 2:** Differences between groups in the study outcomes.

Outcomes	Differences between groups (95% confidence interval)			
	24 weeks	*p* Value	52 weeks	*p* Value
GCF				
Intent-to-treat analysis	.11 (–.01 to .23)	.07†	.17 (.025 to .31)	.02‡
Complete case analysis	.11 (–.01 to .24)	.08†	.17 (.03 to .32)	.02‡
Concentration				
Intent-to-treat analysis	–.012 (–.24 to .21)	.9	.17 (–.1 to .44)	.2
Complete case analysis	.04 (–.2 to .28)	.75	.23 (–.05 to .51)	.1
Reasoning				
Intent-to-treat analysis	.04 (–.15 to .23)	.7	.07 (–.15 to .28)	.5
Complete case analysis	.01 (–.19 to .21)	.9	.056 (–.16 to 27)	.6
Planning				
Intent-to-treat analysis	.21 (–.06 to .48)	.1	.16 (–.13 to .45)	.3
Complete case analysis	.22 (–.08 to .52)	.15	.16 (–.15 to .47)	.3
Memory				
Intent-to-treat analysis	.17 (–.01 to .36)	.07†	.25 (.03 to .47)	.03‡
Complete case analysis	.18 (–.02 to .38)	.08†	.25 (.02 to .48)	.03‡

NOTE. Data presented as *z* scores. Differences between groups calculated as M4 –M2. Calculated from linear mixed effects regression models that included group (M2 or M4), time (baseline, 24 and 52 weeks), and group × time interaction terms.

^†^Trends for differences between groups in estimated mean change from baseline

^‡^Significant differences between groups in estimated mean change from baseline.

Abbreviations: GCF, global cognitive functioning; M2, multiple-modality group; M4, multiple-modality, mind-motor group.

### Secondary analyses

Additional analyses were conducted to understand possible associations between cardiorespiratory fitness (i.e., pVO_2_max) and cognition. At baseline, pVO_2_max was positively associated with GCF (r = .20, p = .006), concentration (r = .24, p = .004), planning (r = .18, p = .02) and memory (r = .17, p = .025), but not reasoning. Following the 24-week intervention period, change in pVO_2_max was positively associated with change in concentration (r = .23, p = .02), but unrelated to change in the remaining outcomes. The association between changes in pVO_2_max and concentration was driven by the M4 group, showing a significant effect (F_(1, 45)_ = 4.8, p = .03, r = .30), whereas the M2 group did not (p = .33). No other associations were observed either at 24 or 52 weeks. Table 3 shows the results of the regression models.

**Table 3:** Associations between cardiorespiratory fitness and study outcomes at baseline and with change scores over time.

Outcomes	pVO_2_max (baseline)	ΔpVO_2_max (24 weeks) ^a^	ΔpVO_2_max (52 weeks) ^b^
GCF	*F*_(1, 120)_ = 7.9, p = .006, r^2^ = .042†	*F*_(1, 101)_ = .85, p = .36, r^2^ = .001	*F*_(1, 88)_ = 2.3, p = .13, r^2^ = .02
Concentration	*F*_(1, 120)_ = 8.5, p = .004, r^2^ = .059†	*F*_(1, 102)_ = 5.8, p = .018, r^2^ = .052†	*F*_(1, 89)_ = 2.1, p = .15, r^2^ = .02
Reasoning	*F*_(1, 120)_ = .92, p = .34, r^2^ = .01	*F*_(1, 102)_ = 1.6, p = .20, r^2^ = .02	*F*_(1, 89)_ = .01, p = .91, r^2^ = .000
Planning	*F*_(1, 120)_ = 5.2, p = .024, r^2^ = .032†	*F*_(1, 102)_ = .03, p = .86, r^2^ = .01	*F*_(1, 89)_ = .003, p = .95, r^2^ = .000
Memory	*F*_(1, 120)_ = 5.1, p = .025, r^2^ = .028†	*F*_(1, 102)_ = .64, p = .43, r^2^ = .01	*F*_(1, 88)_ = .61, p = .43, r^2^ = .007

NOTE: Statistics presented as *F*_change_ and *r*^*2*^_change_ from hierarchical regression models and represent the unique contribution of pVO_2_max to the model, after adjustments for age, gender and years of education.

^a^ Change scores from baseline to 24 weeks.

^b^ Change scores from baseline to 52 weeks.

^†^Significant associations adjusting for age, gender and years of education.

Abbreviations: GCF, global cognitive functioning; pVO_2_max, predicted maximal oxygen consumption

## Discussion

The results of our study did not provide support for the hypothesis that multiple-modality exercise with additional mind-motor training yields greater improvements in cognitive function compared to multiple-modality exercise with additional balance, range of motion and breathing exercises. We did note, however, positive changes over time as result of the intervention. Aligning with previous research in individuals with SCC [36], our results indicated that a 24-weeks of exercise yielded improvements in GCF, concentration, reasoning, planning and memory. Furthermore, additional mind-motor training only demonstrated trends for greater improvements in GCF and memory (both p = .07) at 24 weeks. Even though significant differences between groups were not detected, it is possible that additional 15 minutes of SSE may have positively influenced these outcomes. This partially corroborates previous studies demonstrating that SSE may benefit GCF, attention, mental flexibility [29], memory and executive functioning [30] in cognitively healthy older adults. Compared to those previous studies, the lack of superior effects of the SSE to drive between-group differences in our investigation may be attributed to the short duration and different frequency in which the mind-motor component was administered. Furthermore, other factors may have influenced our results. The current study adopted a RCT design, whereas those previous investigations followed either a quasi-randomized [29] or non-randomized [30] design, which may have resulted in bias. Additionally, discrepancies may have also occurred due to the methodology applied to evaluate cognition in our study (i.e., the CBS battery) compared to the traditional paper-based assessment administered previously [29,30].

In our study, both groups retained the gains in GCF and domain-specific cognitive functioning 28 weeks following the end of the exercise intervention. This is in contrast with the LIFE trial [51], where participants who completed a two-year multicomponent exercise program were not able to retain any gains in cognition after the end of the study. The improved performance within both groups in this study, and particularly in the M4 group, may be partially explained by extraneous factors, such as continuation in self-selected exercise practice or engagement in cognitive training following the end of our intervention. Despite our promising findings, not many studies have investigate the decay of exercise-induced cognitive improvements in older adults after exercise cessation, thus, more research is warranted [17,25].

In our secondary analysis, we sought to explore whether changes in cognition were associated with changes in cardiorespiratory fitness. This set of analyses would allow us to infer a more causal relationship between both exercise interventions and the study outcomes, as observed in previous studies [21,52]. When exploring the cardiovascular outcomes from M4 study in a previous investigation [35], it was observed that both groups demonstrated significant improvements in pVO_2_max after the invention and at 52 weeks, similar to the findings for cognition in the current study. Regardless of these similar changes, no significant associations were found between changes in pVO_2_max and changes in cognition when adjusting for age, gender and years of education, except for concentration at 24 weeks. This suggests that the changes in cognition were not uniquely driven by improvements in fitness, but may have been influenced by other factors. A plausible hypothesis is that such changes may have occurred due to the influence of increased socialization. In fact, social interaction may provide significant cognitive stimulation [51] and partially account for improvements in cognition in older adults [49,53]. Furthermore, in a recent meta-analysis [54] greater effect sizes were observed following exercise in older adults compared to education or no-contact control groups, but not in comparison to active or social engagement control groups [54]. The underlying physiological and neurophysiological changes accountable for improvements in cognition following exercise certainly deserve further investigation particularly in this population of individuals with SCC.

The following limitations should be considered when interpreting the results of our study. Our inclusion criteria may have not been stringent enough to determine the nature, diversity and influence of SCC in cognition function in our sample; a more comprehensive assessment of the SCC would have provided a more homogenous sample. Therefore, our results should be taken carefully. Although the CBS is grounded in well-validated neuropsychological tests [45], this is the first study to apply this method to evaluate the effects of exercise in cognition in older adults. Participants could have also had access to the online version of the CBS and practiced the games before, during or at the end of the study; although we administered an offline version of the CBS, which participants only had accesses during the study assessment period. Nonetheless, if participants accessed the games on their own, this access was most likely at random and would not affect the primary outcome of the study (i.e., differences between groups at 24 weeks). Also, participants included in this study were predominantly Caucasian, well educated, and functionally independent, thus, our results may not be generalizable. In addition, we used a surrogate, although validated, measure of cardiorespiratory fitness (pVO_2_max), which could lack precision in comparison to other more objective measures.

Despite these shortcomings, results from our study indicate that a 24-week, group-based multiple-modality exercise intervention can yield improvements in cognition in older individuals with SCC. Additional mind-motor training only led to trends for greater benefits, particularly in GCF and memory. Future studies could investigate whether individuals presenting additional risk factors for future dementia (e.g., family history of AD, *APOE* ε4 carriers) would respond differently to an exercise intervention similar to what presented in the current study. As well, it is paramount to investigate whether individuals with SCC who engage in regular exercise can reduce the risk of objective cognitive impairment later in life. As indicated by the results of this study, exercise may preserve cognitive function in this population; however more robust evidence in warranted. Also, in the future, including neuroimaging methods to explore changes in brain function (e.g., cortical plasticity) not captured via behavioural data in individuals with SCC would provide a more comprehensive assessment of the effects of exercise in this particular population.

## Abbreviations

AE: aerobic exercise
CBS: Cambridge Brain Sciences cognitive battery
GCF: global cognitive functioning
M2: multiple-modality exercise alone
M4: multiple-modality exercise with additional mind-motor training
MMSE: Mini-Mental State Examination
RCT: randomized controlled trial
SCC: subjective cognitive complaints
SSE: square-stepping exercise
